# Association between advanced lung cancer inflammation index and unstable asthma: a population-based study from the NHANES 2007–2018

**DOI:** 10.3389/fnut.2024.1482328

**Published:** 2024-11-13

**Authors:** Zhou Jin, Wen Sun, Junjun Huang, Guangfa Wang

**Affiliations:** Department of Respiratory and Critical Care Medicine, Peking University First Hospital, Beijing, China

**Keywords:** advanced lung cancer inflammation index, NHANES, population-based study, asthma, cross-sectional study

## Abstract

**Background:**

Asthma exacerbation is associated with obesity and systemic inflammatory diseases, and advanced lung cancer inflammation index (ALI) is a novel biomarker of nutritional inflammation. The purpose of this study was to investigate the potential relationship between ALI and unstable asthma.

**Methods:**

This cross-sectional study utilized data from the 2007–2018 National Health and Nutrition Examination Survey (NHANES). Asthma was assessed through self-reported questionnaires. Multifactorial logistic regression, subgroup analyses, interaction assessments, smoothed curve fitting, and threshold effect analysis models were conducted to investigate the association between ALI and unstable asthma.

**Results:**

The study included 1,822 subjects with current asthma, and we found a linear positive association between ALI and unstable asthma, with higher levels of ALI significantly associated with an increased risk of asthma exacerbations in fully corrected models. However, the associations were not entirely consistent across subgroups. In subgroup analyses by body mass index (BMI) and race, unstable asthma and ALI were independently significant in the BMI (25–29.9) range and the Non-Hispanic White group. Interaction analysis suggested that BMI moderated the relationship between ALI and unstable asthma. Furthermore, smoothed curve fitting showed an inverted U-shaped relationship between log ALI and unstable asthma in subjects with a BMI <25 and male individuals, with inflection points observed at 1.53 and 2.13, respectively.

**Conclusion:**

We found a linear positive association between ALI and unstable asthma, which remained constant in the fully adjusted model. These findings suggest that higher levels of ALI were significantly associated with an increased risk of asthma exacerbation, particularly in asthmatic populations with BMI in the 25–29.9 range. However, more prospective studies are required to confirm our findings.

## 1 Introduction

Asthma is a common chronic respiratory disease worldwide, and its key features are reversible airflow obstruction, airway inflammation, and hyperresponsiveness (1), approximately 300 million individuals worldwide suffer from asthma, including 25 million cases reported in the United States alone (2). According to the Global Asthma Report 2022, more than 1,000 individuals die from asthma each day, and the incidence of asthma is rising at an alarming rate in low- and middle-income countries, placing an enormous burden on global healthcare resources and economies (3, 4). Air pollution, obesity, gender, exposure to several allergens, exposure to tobacco smoke, socioeconomic status, and genetic factors all contribute to the onset and exacerbation of asthma (5–9). The typical asthma symptoms, such as wheezing, coughing, and dyspnea, are often associated with an inflammatory response in the airways, which is influenced by complex interactions between structural and immune cells (5). Among the most serious adverse outcomes of asthma are exacerbations, also known as asthma attacks, which are the leading cause of acute medical visits and hospitalizations among asthma patients (10, 11). Recent studies have shown that blood eosinophils are associated with the inflammatory response in asthma, serving as a potentially important biomarker of asthma exacerbations (12, 13). Research indicates that eosinophils represent a unique subset of cells activated by allergens in the lungs, which may enhance the transition from pro-inflammatory to anti-inflammatory macrophage phenotypes (14). This property suggests their potential role in modulating airway inflammation and recovery. However, eosinophil levels, while valuable, have limitations in sensitivity, particularly in patients with low eosinophil counts, making them less reliable for forecasting exacerbation risk in all patients (12, 15). This highlights the critical need to identify additional biomarkers that are sensitive and specific to asthma exacerbations to improve the management of the disease.

One such promising biomarker is the Advanced lung cancer inflammation index (ALI), a nutritional inflammation biomarker based on body mass index (BMI), serum albumin, neutrophil, and lymphocyte counts, which was first proposed by Jafri et al. (16). ALI was first utilized to assess the systemic inflammation and prognostic status of patients with metastatic non-small cell lung cancer. ALI has also been widely used to assess the prognosis of various tumors, such as colorectal cancer (17–19), hepatocellular carcinoma (20), and gastric cancer (21). In addition to tumors, ALI has also shown potential to predict the long-term outcome and risk of cardiovascular mortality in patients with hypertension (22, 23), to assess the prognosis of Crohn’s disease after surgical resection (24), and to predict coronary artery disease and calcification (25).

Despite the growing body of evidence supporting the use of the ALI in various inflammatory conditions, its relationship with asthma, particularly unstable asthma, has yet to be thoroughly investigated. Considering that asthma and the conditions associated with ALI involve overlapping systemic inflammatory pathways, ALI, as a nutritional and inflammatory biomarker, could be closely linked to obesity, chronic inflammation, and nutritional status in asthma patients (26, 27), suggesting that it may serve as a predictive biomarker for asthma exacerbations. Furthermore, existing studies have predominantly focused on other inflammatory biomarkers, such as eosinophils, leaving a notable gap in the research regarding the association between ALI and asthma. This research gap underscores the necessity to explore the potential application of ALI in asthma management. If ALI is validated as an effective biomarker, it could provide clinicians with more precise management strategies, ultimately improving patient outcomes. Therefore, this study aimed to investigate the relationship between ALI and unstable asthma utilizing data from the National Health and Nutrition Examination Survey (NHANES) from 2007 to 2018.

## 2 Methods

### 2.1 Study population

The data were obtained from NHANES, a nationally representative cross-sectional survey conducted by the Centers for Disease Control and Prevention (CDC) in conjunction with the National Center for Health Statistics (NCHS) (28–30). To obtain a representative sample, a complex and multistage probability whole population sampling methodology was used, which included demographic factors, laboratory tests, physical examinations, and questionnaires. The National Center for Health Statistics NCHS Research Ethics Review Board approved the study protocol (31), and all participants gave informed consent.

**Figure 1:**
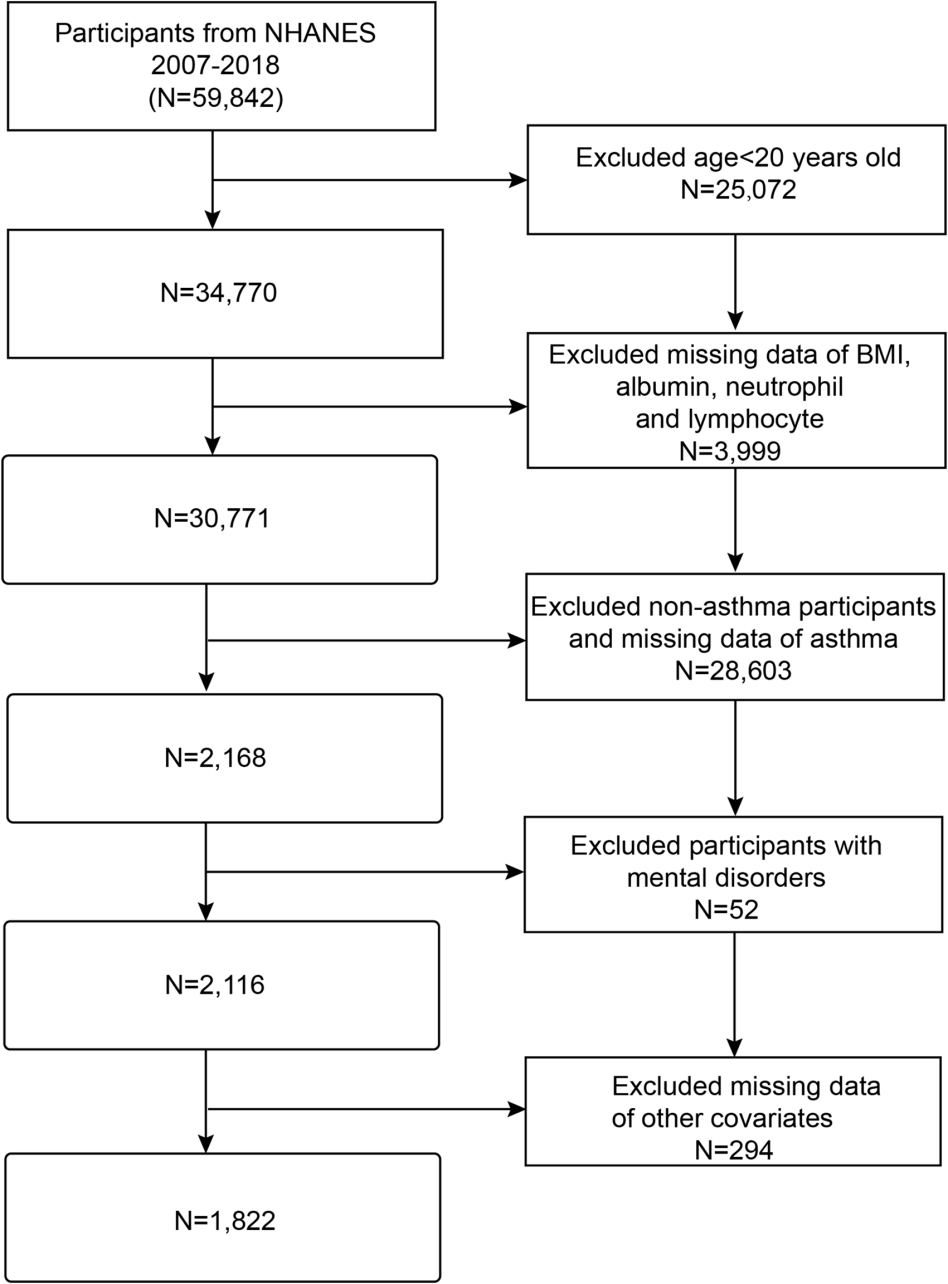
Flow chart of participant’s selection. NHANES, National Health and Nutrition Examination Survey; BMI, Body mass index.

In this study, we analyzed the NHANES database from 2007 to 2018. Initially, 59,842 subjects were enrolled in the study. However, subjects under the age of 20 were excluded (*N* = 25,072), as were individuals with missing data on BMI, serum albumin, neutrophils, and lymphocytes (*N* = 3,999), subjects with non-asthma and missing asthma data (*N* = 28,603), subjects with mental disorders (*N* = 52), and individuals with missing data on other variables (*N* = 294). The final analysis included 1,822 eligible adult participants (Figure 1).

### 2.2 Asthma definition

Subjects included in the final analysis were all current asthmatics, and current asthmatics were categorized as stable or unstable asthma based on whether they had an acute exacerbation or an acute visit to the clinic in the past years, the diagnosis was determined by answering the following four asthma-related questions on the NHANES Medical Condition Questionnaire:

1. Has a doctor or other health professional ever told you that you have asthma?
2. Do you still have asthma?
3. Have you had an asthma attack in the past year?
4. Have you had an emergency care visit for asthma in the past year?

Only those participants who answered yes to questions 1 and 2 were included in the study’s final analysis (32). Stable asthma was defined as both a negative answer to questions 3 and 4, while unstable asthma was defined as a positive answer to either question 3 or 4 (33).

### 2.3 ALI definition

Advanced lung cancer inflammation index was the primary exposure variable in this study, and the ALI was calculated as body mass index (kg/m^2^) × serum albumin (g/dL)/(neutrophil-lymphocyte ratio) (34, 35), where BMI = weight in kilograms/(height in meters)^2^. The ALI was categorized according to tertiles: T1 group (ALI ≤ 51.77), T2 group (51.77 < ALI ≤ 77.81), and T3 group (ALI > 77.81).

### 2.4 Other covariates

Other covariates mainly included sociodemographic characteristics, lifestyle behaviors, long-term conditions, and laboratory indicators. Sociodemographic characteristics included gender (Male, Female), age (years), race (Mexican American, Other Hispanic, Non-Hispanic White, Non-Hispanic Black, and other races), education level (Less than high school, High school or General Educational Development (GED), and Above high school), marital status (Married, Others), family income-to-poverty ratio (PIR), and BMI (kg/m^2^) (BMI was calculated by dividing weight in kilograms by height in meters squared). Lifestyle behaviors included smoking status [never smokers (Smoked <100 cigarettes in life and does not smoke now), former smokers (Smoked at least 100 cigarettes in life and does not smoke now), and current smokers (Smoked at least 100 cigarettes in life and smoke now)] and drinking status (Having at least 12 alcohol drinks per year or not). Long-term conditions hypertension (Yes/No), diabetes (Yes/No). The diagnosis of hypertension was based on a definition of mean systolic blood pressure greater than 130 mmHg or mean diastolic blood pressure greater than 80 mmHg (36, 37), and the physician’s diagnosis of diabetes mellitus was based on a definition of fasting blood glucose ≥7.0 mmol/L or glycosylated hemoglobin ≥6.5% (38). Furthermore, laboratory indicators included albumin (g/dL), neutrophil (1,000 cells/μL), and lymphocyte (1,000 cells/μL). All data and definitions related to these variables are publicly accessible at https://www.cdc.gov/nchs/nhanes/.

### 2.5 Statistical analysis

All statistical analyses were carried out using R (version 4.3) and Empowerstats (version 4.1). The participants were statistically described using the *t*-test or ANOVA for continuous variables and the chi-square test for categorical variables, where data were expressed as mean ± standard deviation (SD) for continuous variables and percentages for categorical variables. The association between ALI and unstable asthma was analyzed using multifactorial logistic regression. There were no covariates adjusted in Model 1. Model 2 adjusted for age, gender, and race. Model 3 adjusted for age, gender, race, education level, marital status, diabetes, hypertension, drinking status, smoking status, and PIR. We utilized generalized weighted smoothed curve fitting and linear models to explore the nonlinear relationship between ALI and unstable asthma, and we log10-transformed the ALI in the smoothed curves to reduce variation caused by bigger data sets (39, 40). Subsequently, further subgroup analyses of age, gender, race, BMI, and PIR, as well as interaction outcome tests, were utilized to examine the association between ALI and unstable asthma in different groups, where age was divided into three groups (20 ≤ Age < 40, 40 ≤ Age < 60, and Age ≥ 60), PIR was divided into three groups (PIR < 1, 1 ≤ PIR < 4, and PIR ≥ 4), and BMl was classified as three groups (BMI < 25 kg/m^2^, 25 kg/m^2^ ≤ BMI < 30 kg/m^2^, and BMI >30 kg/m^2^) according to World Health Organization standards, corresponding to normal weight, overweight, and obesity, respectively. In the subgroup analysis, the model is not adjusted for the stratification variable itself. A threshold effects analysis model was utilized to examine the association and saturation values of unstable asthma and ALI, and the same statistical procedures mentioned above were used for the BMI and gender subgroups. We applied a weighted approach to reduce the data set’s volatility (41, 42). A two-tailed *p* value <0.05 was considered statistically significant.

## 3 Results

### 3.1 Baseline characteristics

The study included 1822 adult participants based on the inclusion and exclusion criteria, with an average age of 49.75 ± 17.44. Among these participants, 33.21% were men, 66.79% were female. The weighted demographic and laboratory data of the participants by asthma exacerbation (759 stable asthma and 1,063 unstable asthma) are shown in Table 1. Unstable asthma participants were more likely to be female, to have a higher BMI and lymphocyte, to have a lower PIR and albumin, and to have higher rates of asthma treatment. According to Table 1, unstable asthma had higher ALI [(72.23) vs. (66.74), *p* = 0.008] in comparison to stable asthma.

**Table 1:** Characteristics of participants enrolled in the study by asthma exacerbation.

	Stable asthma	Unstable asthma	*p* value
	*N* = 759	*N* = 1,063	
Age (years)	49.76 ± 18.38	49.03 ± 16.37	0.386
Gender, *n* (%)			<0.001
Male	315 (41.50%)	311 (29.26%)	
Female	444 (58.50%)	752 (70.74%)	
Race/ethnicity, *n* (%)			0.319
Mexican American	76 (10.01%)	94 (8.84%)	
Other Hispanic	64 (8.43%)	111 (10.44%)	
Non-Hispanic White	335 (44.14%)	493 (46.38%)	
Non-Hispanic Black	205 (27.01%)	254 (23.89%)	
Other races	79 (10.41%)	111 (10.44%)	
Education level, *n* (%)			0.139
Less than high school	150 (19.76%)	245 (23.05%)	
High school or GED	190 (25.03%)	234 (22.01%)	
Above high school	419 (55.20%)	584 (54.94%)	
Marital status, *n* (%)			0.094
Married	334 (44.01%)	426 (40.08%)	
Others	425 (55.99%)	637 (59.92%)	
Diabetes, *n* (%)			0.078
Yes	140 (18.45%)	232 (21.83%)	
No	619 (81.55%)	831 (78.17%)	
Hypertension, *n* (%)			0.484
Yes	343 (45.19%)	498 (46.85%)	
No	416 (54.81%)	565 (53.15%)	
Asthma treatment, *n* (%)			<0.001
Yes	78 (10.28%)	230 (21.63%)	
No	681 (89.72%)	833 (78.37%)	
Drinking status, *n* (%)			0.682
Yes	541 (71.28%)	767 (72.15%)	
No	218 (28.72%)	296 (27.85%)	
Smoking status, *n* (%)			0.344
Never	385 (50.77%)	518 (48.76%)	
Former	205 (27.05%)	278 (26.12%)	
Now	169 (22.18%)	267 (25.13%)	
PIR	2.32 ± 1.61	2.07 ± 1.59	<0.001
BMI (kg/m^2^)	30.53 ± 8.00	32.00 ± 9.06	<0.001
Albumin (g/dL)	4.15 ± 0.35	4.11 ± 0.37	0.002
Neutrophil (1,000 cells/μL)	4.56 ± 1.88	4.62 ± 1.89	0.344
Lymphocyte (1,000 cells/μL)	2.22 ± 1.43	2.27 ± 0.85	0.028
ALI	66.74 ± 46.64	72.23 ± 42.23	0.008

Mean + SD for continuous variables: the *p* value was calculated by the weighted linear regression model. (%) for categorical variables: the *p* value was calculated by the weighted chi-square test.

ALI, Advanced lung cancer inflammation index; PIR, Family income-to-poverty ratio; BMI, Body mass index.

The study population was divided into three groups based on the tertile of ALI values (T1, *n* = 607, ALI ≤ 51.77; T2, *n* = 607, 51.77 < ALI ≤ 77.81; T3, *n* = 608, ALI > 77.81) are shown in Table 2. Participants with higher ALI were more likely to be younger, non-Hispanic Black, diabetic, hypertensive, have a higher BMI, albumin, and lymphocyte, and have lower neutrophil levels compared to the lowest ALI tertile. Furthermore, those with higher ALI had higher rates of asthma attacks (Table 2).

**Table 2:** Weighted basic characteristics of participants by ALI.

	Overall	T1	T2	T3	*p* value
	*N* = 1822	*N* = 607	*N* = 607	*N* = 608	
Age (years)	49.75 ± 17.44	52.17 ± 18.49	47.24 ± 16.71	48.59 ± 16.06	<0.001
Gender, *n* (%)					0.159
Male	605 (33.21%)	218 (35.93%)	201 (33.14%)	186 (30.63%)	
Female	1,217 (66.79%)	389 (64.07%)	406 (66.86%)	422 (69.37%)	
Race/ethnicity, *n* (%)					<0.001
Mexican American	170 (9.33%)	54 (8.90%)	64 (10.54%)	52 (8.55%)	
Other Hispanic	175 (9.60%)	50 (8.24%)	62 (10.21%)	63 (10.36%)	
Non-Hispanic White	828 (45.44%)	322 (53.05%)	301 (49.59%)	205 (33.72%)	
Non-Hispanic Black	459 (25.19%)	99 (16.31%)	129 (21.25%)	231 (37.99%)	
Other races	190 (10.43%)	82 (13.51%)	51 (8.40%)	57 (9.38%)	
Education level, *n* (%)					0.677
Less than high school	395 (21.68%)	137 (22.57%)	131 (21.58%)	127 (20.89%)	
High school or GED	424 (23.27%)	142 (23.39%)	131 (21.58%)	151 (24.84%)	
Above high school	1,003 (55.05%)	328 (54.04%)	345 (56.84%)	330 (54.28%)	
Marital status, *n* (%)					0.112
Married	867 (47.59%)	303 (49.98%)	296 (48.83%)	268 (44.11%)	
Others	955 (52.41%)	304 (50.02%)	311 (51.17%)	340 (55.89%)	
Diabetes, *n* (%)					0.041
Yes	280 (15.37%)	80 (13.20%)	88 (14.53%)	112 (18.40%)	
No	1,542 (84.63%)	527 (86.80%)	519 (85.47%)	496 (81.60%)	
Hypertension, *n* (%)					<0.001
Yes	841 (46.16%)	272 (44.81%)	246 (40.53%)	323 (53.12%)	
No	981 (53.84%)	335 (55.19%)	361 (59.47%)	285 (46.88%)	
Drinking status, *n* (%)					0.264
Yes	1,386 (76.07%)	469 (77.23%)	469 (77.22%)	448 (73.63%)	
No	436 (23.93%)	138 (22.77%)	138 (22.78%)	160 (26.37%)	
Smoking status, *n* (%)					0.056
Never	902 (49.51%)	286 (47.05%)	325 (53.62%)	291 (47.80%)	
Former	486 (26.67%)	159 (26.27%)	149 (24.56%)	178 (29.25%)	
Now	434 (23.82%)	162 (26.69%)	133 (21.82%)	139 (22.95%)	
Stable asthma, *n* (%)					0.006
Yes	780 (42.81%)	291 (47.95%)	242 (39.91%)	247 (40.64%)	
No	1,042 (57.19%)	316 (52.05%)	365 (60.09%)	361 (59.36%)	
PIR	2.17 ± 1.60	2.18 ± 1.62	2.22 ± 1.65	2.12 ± 1.54	0.582
BMI (kg/m^2^)	31.96 ± 8.80	27.90 ± 6.76	31.41 ± 8.15	35.53 ± 9.41	<0.001
Albumin (g/dL)	4.13 ± 0.36	4.09 ± 0.39	4.15 ± 0.37	4.13 ± 0.33	0.019
Neutrophil (1,000 cells/μL)	4.59 ± 1.89	5.63 ± 2.02	4.56 ± 1.51	3.67 ± 1.38	<0.001
Lymphocyte (1,000 cells/μL)	2.25 ± 1.13	1.80 ± 0.58	2.23 ± 0.58	2.77 ± 1.44	<0.001

Mean + SD for continuous variables: the *p* value was calculated by the weighted linear regression model. (%) for categorical variables: the *p* value was calculated by the weighted chi-square test.

ALI, Advanced lung cancer inflammation index; PIR, Family income-to-poverty ratio; BMI, Body mass index.

### 3.2 Relationship between ALI and unstable asthma

Table 3 shows the results of the multivariate logistic regression analyses of the three models for the association between ALI and unstable asthma. There was a significant positive association between ALI and unstable asthma, which was shown to be statistically significant in all three models, and participants with higher levels of ALI had a 5% increased risk of asthma exacerbation in the fully calibrated model (OR:1.05, 95% CI: 1.04–1.05). When ALI was divided into tertiles, patients in the highest tertile had a 15% increased risk of asthma exacerbation compared to those in the lowest tertile (OR: 1.15, 95%CI: 1.13, 1.17) (Table 3).

**Table 3:** Odds ratios for the association between ALI and unstable asthma.

Exposure	Model 1: OR (95%CI), *p*	Model 2: OR (95%CI), *p*	Model 3: OR (95%CI), *p*
ALI	1.06 (1.05, 1.06)	1.06 (1.05, 1.06)	1.05 (1.04, 1.05)
	<0.001	<0.001	<0.001
ALI classification			
Tertiles 1	Reference	Reference	Reference
Tertiles 2	1.33 (1.31, 1.35)	1.31 (1.29, 1.34)	1.29 (1.27, 1.31)
	<0.001	<0.001	<0.001
Tertiles 3	1.24 (1.22, 1.26)	1.24 (1.22, 1.26)	1.15 (1.13, 1.17)
	<0.001	<0.001	<0.001
*p* for trend	<0.001	<0.001	<0.001

Model 1: No covariates were adjusted.

Model 2: Age, gender, and race were adjusted.

Model 3: Age, gender, race, education level, marital status, diabetes, hypertension, drinking status, smoking status, and PIR were adjusted.

ALI: Advanced lung cancer inflammation index; PIR, Family income-to-poverty ratio.

### 3.3 Subgroup analysis

To ensure that our results were robust across groups, we conducted subgroup analyses and interaction outcome tests based on age, gender, BMI, race, and PIR status (Table 4). Subgroup analysis revealed that the association between ALI and unstable asthma was inconsistent across subgroups. In subgroup analyses by BMI, ALI and unstable asthma were independently significant in the BMI (25–29.9) range. Interaction tests showed that BMI moderated the association between ALI and unstable asthma (*p* for interaction <0.05). In subgroup analyses by race, ALI and unstable asthma were independently significant in the Non-Hispanic White group. However, there was no independent significant relationship between ALI and unstable asthma in any of the subgroup analyses by gender, age, or PIR.

**Table 4:** Subgroup analysis of the association between ALI and unstable asthma.

Subgroup	OR(95%CI), *p*	*p* for interaction
Gender		0.877
Male	1.05 (0.98, 1.12), 0.145	
Female	1.04 (0.98, 1.11), 0.168	
Age (years)		0.714
20–39	1.07(0.98, 1.17), 0.121	
40–59	1.02(0.94, 1.11), 0.649	
≥60	1.05(0.98, 1.12), 0.123	
BMI (kg/m^2^)		0.022
<25	0.95(0.85, 1.06), 0.360	
25–29.9	1.14(1.04, 1.26), 0.006	
≥30	1.01(0.95, 1.08), 0.765	
Race/ethnicity		0.374
Mexican American	0.95(0.78, 1.16), 0.617	
Other Hispanic	0.97(0.80, 1.18), 0.777	
Non-Hispanic White	1.08(1.01, 1.15), 0.022	
Non-Hispanic Black	1.01(0.94, 1.09), 0.754	
Other Races	1.15(0.97, 1.35), 0.107	
PIR		0.351
<1	1.09(0.99, 1.19), 0.065	
1–3.9	1.02(0.96, 1.08), 0.562	
≥4	1.08(0.98, 1.19), 0.143	

Age, gender, race, education level, marital status, diabetes, hypertension, drinking status, smoking status, and PIR were adjusted. In the subgroup analysis, the model is not adjusted for the stratification variable itself.

ALI, Advanced lung cancer inflammation index; PIR, Family income-to-poverty ratio; BMI, Body mass index.

### 3.4 Analysis of nonlinear and saturation effects between ALI and unstable asthma

**Figure 2:**
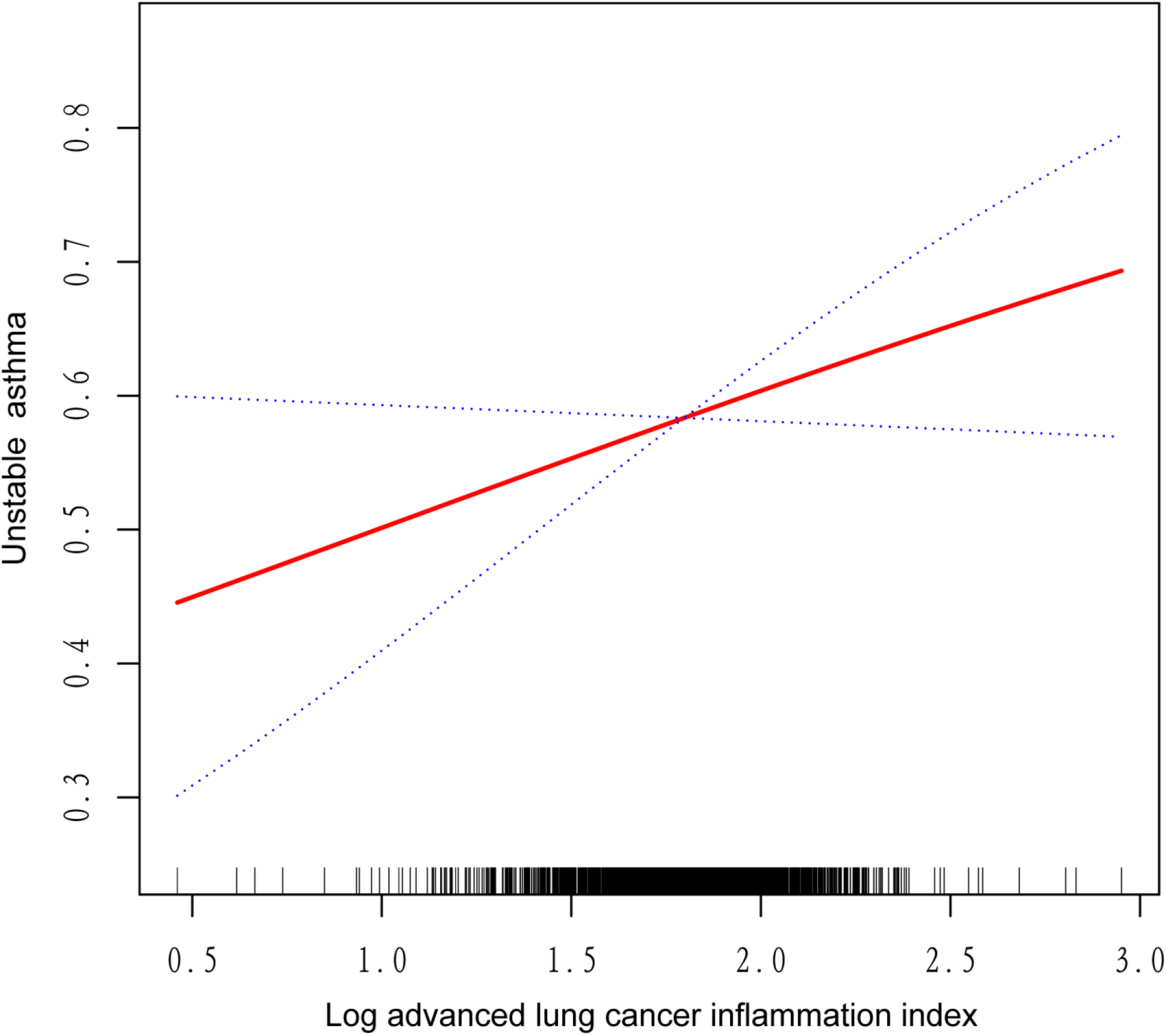
The linear associations between log advanced lung cancer inflammation index and unstable asthma. The solid red line represents the smoothing curve fit between variables. Blue bands represent the 95% confidence interval from the fit.

**Figure 3:**
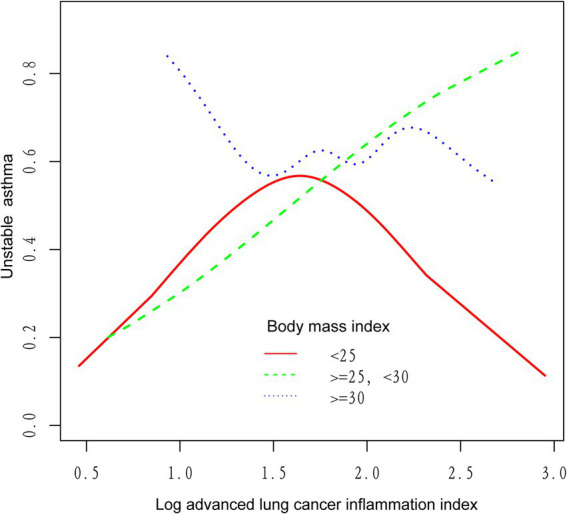
The association between log advanced lung cancer inflammation index and unstable asthma stratified by body mass index. The red line represents the BMI < 25, the green line represents the 25 ≤ BMI < 30, and the blue line represents the BMI ≥ 30.

**Figure 4:**
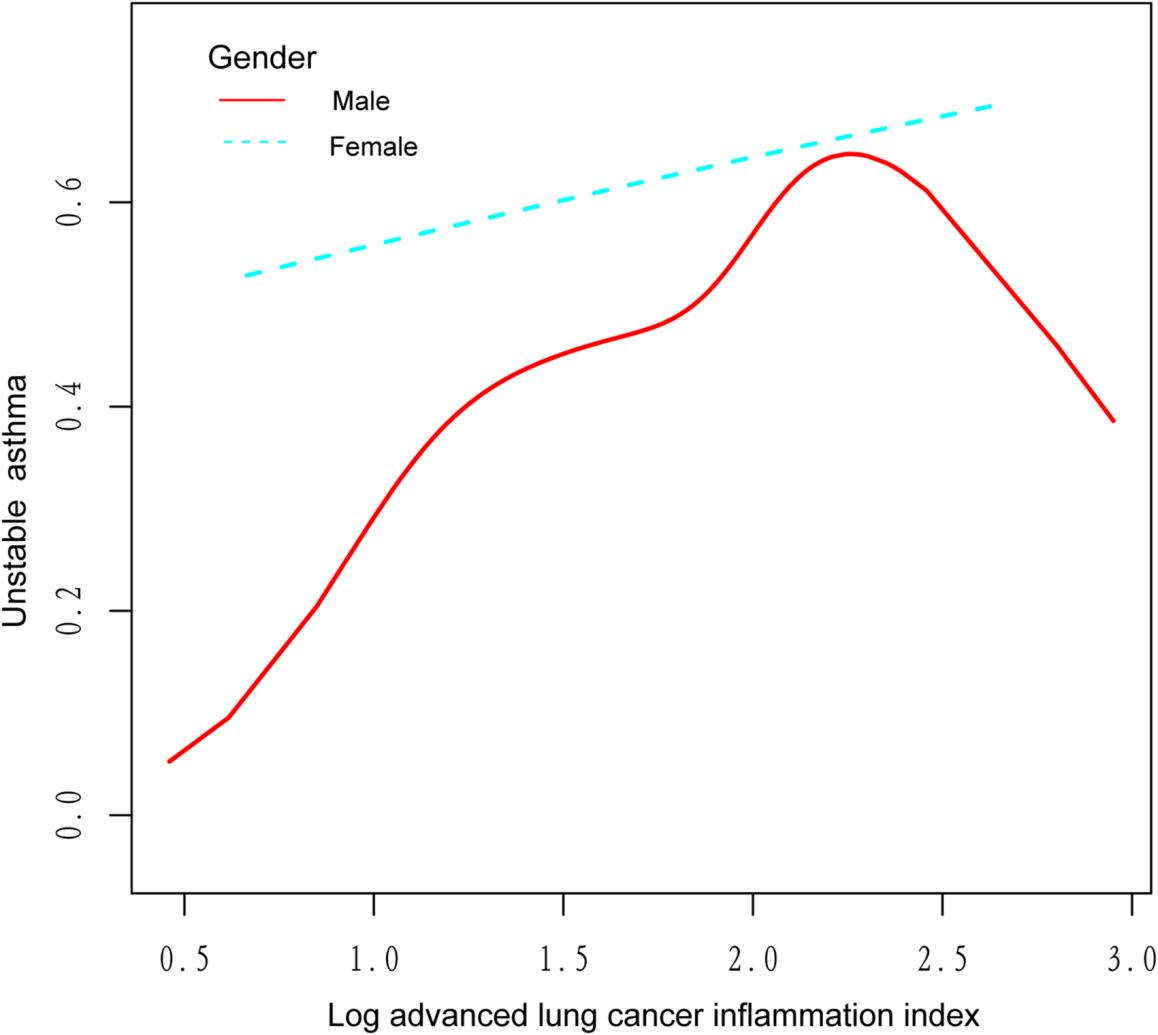
The association between log advanced lung cancer inflammation index and unstable asthma stratified by gender. The red line represents the male and the blue line represents the female.

We utilized a weighted generalized model with smooth curve fitting to describe the nonlinear relationship and saturation effect of log ALI and unstable asthma. The results found a linear positive relationship between log ALI and unstable asthma (Figure 2). Notably, we found an inverted U-shaped relationship between log ALI and unstable asthma in the BMI < 25 intervals (Figure 3), with an inflection point of 1.53 by a two-band linear regression model. Specifically, the risk of asthma exacerbation increased with increasing ALI at the front end of the inflection point [40.36 (39.88, 40.84), *p* < 0.001], while decreasing with increasing ALI at the back end of the inflection point [0.12 (0.12, 0.12), *p* < 0.001] (Table 5). Furthermore, when stratified by gender, we found an inverted U-shaped relationship between unstable asthma and log ALI in the male population (Figure 4), with a two-band linear regression model suggesting an inflection point of 2.13 (Table 5).

**Table 5:** Threshold effect analysis of ALI on unstable asthma using a two-piecewise linear regression model.

Unstable asthma	Adjusted OR (95% CI)
	*p* value
ALI	
Inflection point	2.16
<2.16	1.45 (1.25, 1.69) < 0.001
>2.16	0.49 (0.24, 1.02) 0.057
Log likelihood ratio	0.007
BMI < 25	
Inflection point	1.53
<1.53	40.36 (39.88, 40.84) < 0.001
>1.53	0.12 (0.12, 0.12) < 0.001
Log likelihood ratio	<0.001
25 ≤ BMI < 30	
Inflection point	1.37
<1.37	10.08 (9.87, 10.30) < 0.001
>1.37	3.45 (3.43, 3.46) <0.001
Log likelihood ratio	<0.001
Male	
Inflection point	2.13
<2.13	2.04 (1.59, 2.62) <0.001
>2.13	0.31 (0.12, 0.80) 0.016
Log likelihood ratio	<0.001
Female	
Inflection point	1.97
<1.97	0.94 (0.74, 1.20) 0.630
>1.97	2.42 (1.38, 4.24) 0.002
Log likelihood ratio	0.007

Age, race, education level, marital status, diabetes, hypertension, drinking status, smoking status, and PIR were adjusted.

ALI, Advanced lung cancer inflammation index; PIR, Family income-to-poverty ratio; BMI, Body mass index.

## 4 Discussion

In this study, we investigated the association between ALI and unstable asthma. Our findings suggest a linear positive relationship between ALI and unstable asthma. After correcting for relevant confounders, we found that higher levels of ALI were significantly associated with an increased risk of asthma exacerbation. However, the relationship between ALI and unstable asthma was not entirely consistent across subgroups, and in subgroup analyses by BMI and race, unstable asthma and ALI were independently significant in the BMI (25–29.9) range and in the Non-Hispanic White group. Interaction outcome tests showed that BMI moderated the relationship between ALI and unstable asthma. Notably, smoothed curve fitting suggested an inverted U-shaped relationship between log ALI and unstable asthma in the BMI < 25 interval and male population, with inflection points at 1.53 and 2.13, respectively, according to the two-band linear regression model. Our findings suggest that the level of ALI in the assessment of asthma exacerbations may have potential clinical value, particularly in asthmatic populations with BMI in the 25–29.9 range. To our knowledge, this is the first study to investigate the relationship between ALI and unstable asthma.

Asthma is a heterogeneous disease typically characterized by airway obstruction due to chronic inflammation (27), with infiltration and activation of immune cells such as eosinophils, neutrophils, lymphocytes, mast cells, dendritic cells, and macrophages, which together lead to excessive inflammation of the airways (43). These eosinophils are driven by T helper 2 (Th2) cells, which combine with cytokines produced by Th2 cells, such as interleukin-5 (IL-5), to activate an inflammatory response in the airways (44). Therefore, eosinophils are an important biomarker of asthma. However, eosinophil-activated inflammatory responses are only applicable to asthmatics with a type 2 (T2) inflammatory response. Predicting the risk of asthma attacks in patients with non-T2 types is not effective (45). Wills-Karp et al. proposed that neutrophils exacerbate asthma attacks by co-mediating inflammatory responses with Th17 cells (46). Radermecker et al. (47) found that environmental risk factors promote allergic asthma by recruiting pulmonary neutrophils. Furthermore, numerous studies have found that obesity is a risk factor for asthma attacks (48, 49), with obese asthmatics having higher blood neutrophil counts than non-obese asthmatics (50). The study also found that patients with unstable asthma had higher BMI levels.

Based on BMI, serum albumin, neutrophil, and lymphocyte counts, ALI was initially widely utilized to assess the prognosis of advanced lung cancer as a novel biomarker of nutritional inflammation (16, 51, 52). Kusunoki et al. (24) found that ALI predicted postoperative recurrence after surgical resection of chronic inflammatory bowel illness (Crohn’s disease). Yuan et al. (53) found that higher ALI levels on admission were strongly associated with lower all-cause and cardiovascular mortality in elderly patients with heart failure (HF) and were independently predictive for evaluating long-term mortality from HF in elderly patients. Chen et al. (54) studied 3,888 patients with Type 2 diabetes mellitus (T2DM) and found that elevated levels of ALI in T2DM patients were strongly associated with reduced all-cause and cardiovascular mortality. However, no previous studies have investigated the relationship between ALI and asthma exacerbations.

In this study, we found a significant positive association between ALI levels and unstable asthma, even after controlling for all confounding variables. However, the relationship between ALI and unstable asthma was not entirely consistent across subgroups. In BMI-adjusted subgroup analyses, ALI and unstable asthma were independently significant in the BMI (25–29.9) range rather than in the obese (BMI > 30) range, which may be because obese people are in a chronic inflammatory state for a long time, and the body may gradually adapt to this state through certain mechanisms, resulting in a relatively weakened effect of inflammation on the course of asthma, whereas overweight patients with a BMI of 25–29.9 are still in the pre-obesity stage, and may not have developed such an adaptive mechanism (55, 56). Therefore, the effect of inflammatory index (ALI) on asthma exacerbation may be more significant in overweight patients. Furthermore, BMI was found to modulate the association between ALI and unstable asthma. Previous studies have shown that increased BMI levels are positively correlated with airway wall thickness and airway adipose tissue, the accumulation of which stimulates chronic low-grade systemic inflammation and alters immune system function, leading to excessive airway narrowing and asthma attacks (57, 58). Therefore, ALI, a novel BMI-based biomarker of nutritional inflammation, has potential clinical value in assessing the risk of asthma attacks. Notably, we found an inverted U-shaped relationship between log ALI and unstable asthma in BMI <25 range populations, with an inflection point of 1.53 by a two-band linear regression model, indicating that healthy weight asthmatics have opposite risks of asthma attacks at the two ends of the inflection point of 1.53.

There are several limitations in this study. First, this study was an associational cross-sectional study. Therefore, it was not possible to determine a causal relationship between ALI and unstable asthma. Secondly, differentiating between T2 asthma and non-T2 asthma with the level of ALI was not possible.

Thirdly, asthma diagnosis in our study was based on self-reported questionnaires, which may not be as accurate as clinical diagnoses. Furthermore, asthma attacks could be confounded by other conditions that also cause wheezing, potentially introducing bias into the analysis, and there is a possibility of both selection bias and recall bias, which could influence the study’s findings. Besides, the NHANES dataset does not capture information on the annual frequency of asthma exacerbations, limiting our ability to stratify ALI levels according to asthma severity. Since our study was limited to asthma patients in the United States, it is important to validate these results in a control sample of real patients from a more diverse population to ensure the generalizability of our conclusions. Finally, despite adjusting for several relevant confounders, we were unable to exclude other confounders with potential impacts. Despite these limitations, this study has several advantages. To our knowledge, it is the first study to investigate the relationship between ALI and unstable asthma, emphasizing the potential clinical value of ALI as a novel nutrient-inflammation biomarker in asthma exacerbations. Moreover, the research utilizes a nationwide multiethnic representative sample, allowing us to analyze the association more systematically. For the future direction, longitudinal studies are needed to explore the causal relationship between ALI and asthma exacerbations. Additionally, investigating the association between ALI and varying severities of asthma could provide valuable insights, potentially enhancing the clinical management of asthma. Furthermore, more prospective studies with large samples on the relationship between ALI and unstable asthma are also needed to confirm our findings.

## 5 Conclusion

We found a linear positive association between ALI and unstable asthma, which continued stable in the fully corrected model. Interaction outcome tests found that BMI moderated the relationship between ALI and unstable asthma, and unstable asthma and ALI were independently significant in the BMI (25–29.9) range. Furthermore, an inverted U-shaped relationship was found between log ALI and asthma exacerbations in the BMI < 25 range, with an inflection point of 1.53. These results indicate that higher levels of ALI were significantly associated with an increased risk of asthma exacerbation, particularly in asthmatic populations with BMI in the 25–29.9 range, providing early warning to high-risk individuals. This has important implications for everyday medical practice, as clinicians may consider incorporating ALI measurements into routine evaluations of asthma patients, especially those with specific BMI ranges. By identifying high-risk individuals early, tailored interventions and closer monitoring can be implemented, potentially improving outcomes for patients prone to asthma exacerbations. However, more prospective studies are required to confirm our findings.

## Data Availability

The datasets presented in this study can be found in online repositories. The names of the repository/repositories and accession number(s) can be found below: the survey data are publicly available online for data users and researchers worldwide (www.cdc.gov/nchs/nhanes/).
